# Acute Response to Training after Returning from the Off-Season in Elite Rugby League Athletes

**DOI:** 10.5114/jhk/185442

**Published:** 2024-04-25

**Authors:** Ryan Simmons, Anthony Leicht, Wade Sinclair, Paul Bowman, Michael Dobbin, Kenji Doma

**Affiliations:** 1 Sport and Exercise Science, College of Healthcare Sciences, James Cook University, Townsville, Australia.; 2 North Queensland Cowboys Rugby League Football Club, Townsville, Australia.; 3 Western Australian Institute of Sport, Perth, Australia.; 4 Orthopeadic Institute of Queensland, Townsville, Australia.

**Keywords:** myalgia, creatine kinase, rugby, training loads

## Abstract

The purposes of this study were to quantify the physiological response to the initial two-week preseason period in elite male rugby league (RL) athletes, and to determine if a repeated bout effect (RBE) occurs. Eighteen RL players were monitored for the initial two-week preseason period. Blood samples were collected on days (D)1, D2, D4, D5, D8, D9, D11 and D12 to measure creatine kinase (CK). Neuromuscular power was assessed on D1, D5, D8 and D12. During field-based sessions, the external training load was quantified using global positioning system technology, whilst the internal load was quantified using the training impulse and the session rating of perceived exertion. Resistance-based gym session volume was quantified by total repetitions x weight lifted. Perceived measures of fatigue and muscle soreness were assessed on all training days. Two-way (day x week) repeated measures analysis of variance and Bonferroni’s corrected post-hoc tests identified significant changes. There were no significant changes in CK activity (649.2 ± 255.0 vs. 673.8 ± 299.1 µL; p = 0.63) or internal training load measures from week 1 to week 2. External training load measures including total distance (4138.1 ± 198.4 vs. 4525.0 ± 169.2 m; p < 0.001) and repeated high-intensity efforts (12.6 ± 1.8 vs. 17.5 ± 1.8 au; p < 0.001) significantly increased in week 2 compared to week 1. Internal training loads and CK activity did not change in response to an increase in external training loads during the initial preseason. The current results provide support for a ‘real world’ perspective of the RBE phenomenon that may be more applicable for team sport practitioners.

## Introduction

Rugby league (RL) is a collision-based intermittent team sport played at amateur, semi-elite and elite levels. Players are exposed to periods of high intensity activity interspersed with periods of low intensity activity (Johnston et al., 2014). Thus, RL players are required to complete various modes of training at high intensities to physically prepare them for the demands of competition. The elite competition in Australia and New Zealand is the National Rugby League (NRL). Like many team sports, the NRL season comprises three macro-cycles in the following order: the off-season, the preseason and the competition season (Gamble, 2006). Each of these phases has separate goals related directly to players’ physical fitness and wellbeing (Buchheit et al., 2013). For example, the off-season occurs between the end of the competition season (September–October) and the beginning of the preseason (November) and is an opportunity for players to recuperate from the preceding competition season. Conversely, the preseason phase aims to optimise physical and technical qualities of players, whilst the focus of the competition phase is to maintain these qualities (Jeong et al., 2011). Of these phases, athletes are exposed to the greatest training loads in the preseason (Jeong et al., 2011), which has been known to cause exercise induced muscle damage (EIMD) (Simmons et al., 2021).

The signs and symptoms of EIMD include increased intramuscular enzymes in the blood (e.g., creatine kinase, CK) and perceived muscle soreness (Doma et al., 2015). The EIMD symptoms are also known to impair muscle force-producing capabilities for several days after a training stimulus (Hyldahl and Hubal, 2014), potentially impacting the athletes’ ability to perform at the required capacity. However, repeated exposure to a training stimulus was reported to ameliorate markers of EIMD and is termed the ‘repeated bout effect’ (RBE) (Hyldahl et al., 2017; Hyldahl and Hubal, 2014; McHugh et al., 1999). Declines in CK across two time points of similar training loads indicative of an RBE have been reported for athletes in team sports such as handball (Concepcion-Huertas et al., 2013), American football (Kraemer et al., 2013) and futsal (Miloski et al., 2016). However, these RBEs were observed across the final weeks of the preseason (Miloski et al., 2016) and competition (Concepcion-Huertas et al., 2013; Kraemer et al., 2013) periods with the duration between data collection varying from two to 12 weeks. Collecting data sporadically makes it challenging to determine the acute physiological response to a specific training stimulus in a single macro-cycle such as the preseason. Furthermore, only one of the aforementioned studies (Kraemer et al., 2013) involved participants from a contact sport. Athletes participating in contact sports experience significant impact-induced muscle damage (IIMD) as a result of physical collisions with the ground and opposition players (Naughton et al., 2018), which exhibits similar symptoms to those of EIMD and may affect the occurrence of the RBE phenomenon. Thus, athletes in contact sports should have their physical impact monitored in both training and competition to gain a more holistic understanding of the external training load.

The optimisation of training loads in the preseason can have a significant impact on the availability of team sport athletes to train and compete throughout the competition phase of the year. For example, higher preseason training loads were associated with increased training and match availability during the competitive season (Gabbett, 2016; Windt et al., 2017). A greater understanding of the physiological responses to training during the initial preseason period may assist in optimising readiness for each training session, reduce the risk of overtraining and enhance training quality throughout the remainder of the preseason period (Podgórski et al., 2021). To the authors’ knowledge, no studies have investigated the physiological response to training during a NRL preseason. Given the unique physical demands of elite RL, it is important to understand the physiological response to the initial weeks or micro-cycles of the preseason period to ensure training loads can be optimised and enhance athletes’ preparation for the latter preseason and the upcoming competition season. Therefore, the purposes of this study were to: 1) quantify the EIMD (CK activity, muscle soreness, neuromuscular performance) response experienced during the initial two weeks of the preseason phase in elite male RL athletes; and 2) determine whether an RBE occurred during the study period.

## Methods

### Participants

A group of 18 elite, male, RL players from the same NRL club participated in the study during the preseason leading into the 2020 NRL competition season. Prior to the commencement of the study, players received comprehensive explanations of the study and provided voluntary written informed consent. Players reported no illness, injury or medication that would contraindicate any of the testing and training protocols and were familiar with testing protocols and wearable technologies as part of their normal training and monitoring routines. To minimise biological variation, players refrained from strenuous activity for at least 48 h prior to the initial testing session and maintained their dietary habits throughout the study period. Ethics approval from the Institutional Review Board of the James Cook University (protocol code: H7918; approval date: 18 October 2019) was obtained prior to the recruitment of players in line with the Declaration of Helsinki.

### Design and Procedures

**Figure 1:**
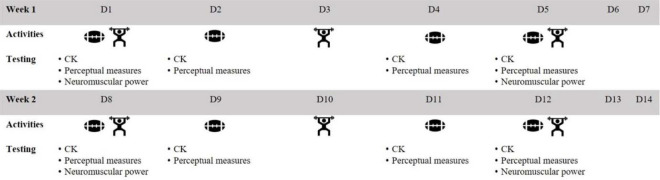
Schematic outline of the schedule of training and testing throughout the study period. CK: creatine kinase, D: day.

A repeated measures design was used to assess the acute responses (i.e., CK activity, perceptual measures, neuromuscular power, the heart rate) to the initial period of the preseason phase. Players participated in eight field-based skill/conditioning sessions in the morning and six resistance-based gym sessions in the afternoon over two weeks (Figure 1). Both upper and lower body compound exercises were included in each resistance-based session, involving concentric and eccentric contractions at moderate-high intensities (80–90% of the one repetition maximum). The strength training volume was augmented at the beginning of each week, given that athletes were expected to tolerate a higher volume of training after a weekend of rest. The field-based skill/condition sessions consisted of multiple running activities at, or slightly above, maximal aerobic speed, as well as fundamental skills (catching, passing and tackling on soft bags) and small-sided games. Capillary blood samples were collected on day 1 (D1), 2 (D2), 4 (D4), 5 (D5), 8 (D8), 9 (D9), 11 (D11) and 12 (D12) at the same time each morning prior to the commencement of training, with D1–D5 occurring during the first week of the pre-season and D8–D12 at the second week of the pre-season. Upper and lower body neuromuscular performance was assessed on D1, D5, D8 and D12. Lower body neuromuscular performance was assessed in the morning prior to the field-based session, whilst upper body neuromuscular performance was assessed in the afternoon prior to the resistance-based gym session. These neuromuscular performance tests were separated between morning and afternoon to establish the readiness to train, as field-based sessions primarily involved exercises requiring the lower body, whilst resistance-based sessions consisted of upper body exercises. The neuromuscular performance assessments were conducted on D1 (beginning of the first week) to establish baseline values, on D5 and D12 (end of each week) to determine the accumulation of training-induced stress during the first and the second week, and D8 (beginning of week 2) to determine the athletes’ recovery status following a weekend of rest from the first week of training. Furthermore, all athletes undertook standardised cold-water immersion as a recovery modality following each field-based session, to enhance training readiness for the afternoon. All data were entered into an athlete management system (Smartabase, Fusion Sport, Brisbane, Australia) either manually or via an application programming interface for collation and subsequent analysis.

### Measures

#### Muscle Damage Marker

Fingertip capillary blood samples (30 μL) were collected from each player for analysis of CK activity and stored in a heparinised microtube in a cool environment (i.e., foam container with ice blocks). Within 30 min after collection, each blood sample was placed on a measurement strip using a pipette, and analysed via a Reflotron Plus system (Roche Diagnostics, Grenzacherstrasse, Switzerland), which has previously been reported as a valid and reliable tool for CK assessment (Horder et al., 1991).

#### Internal Training Load

Perceptual measures of fatigue and muscle soreness were assessed on each training day morning prior to or upon arriving at the training facility (Figure 1). Players were asked to rate each measure on a Likert scale from 1 to 10 with 10 being the best possible score (i.e., no fatigue or muscle soreness). Players completed questionnaires via an athlete management system mobile application (Smartabase Athlete, Fusion Sport, Brisbane, Australia). The internal load of field-based sessions was quantified via fitted heart rate monitors (Firstbeat Sports, Firstbeat, Finland) with a sampling frequency of 1000 Hz for heartbeat. The training impulse (TRIMP) (Stagno et al., 2007) was calculated from the heart rate recordings with a low measurement error previously reported for the TRIMP (coefficient of variation of ~5%) (Turner et al., 2017). Each player wore the same monitor throughout the duration of the study to minimise between-monitor error (Bogdány et al., 2016). Internal loads for all field-based sessions were also quantified via a session rating of perceived exertion (sRPE) as previously defined (Foster et al., 2001).

#### External Training Load

The external load was quantified during each field-based skill/conditioning session using a 10-Hz global positioning system (GPS). The GPS device (Vector S7, Catapult, Melbourne, Australia) also consisted of an accelerometer and was worn in a fitted harness between the scapulae of the player. Scott and colleagues (2016) have reported strong validity and reliability of 10-Hz GPS devices in measuring distances covered at both low and high velocities and each player wore the same device for each training session to minimise interunit error (Scott et al., 2016). At the conclusion of each session, data were downloaded and analysed using the proprietary software (Openfield, Catapult, Melbourne, Australia). The cumulative session totals for the following metrics per player were then exported into a customised Microsoft Excel spreadsheet (Microsoft, Redmond, USA): total distance (m), high velocity (>15 km/h) distance (m), PlayerLoadTM (arbitrary units, AU), repeat high-intensity efforts (RHIEs), and the count of body impacts cumulatively in three bands (B4 = 7–8 G, B5 = 8–10 G, B6 ≥ 10 G). The external load during resistance-based gym sessions was quantified using the following equation: total repetitions x weight lifted = total resistance volume (Hiscock et al., 2015).

#### Neuromuscular Function

Lower body neuromuscular performance was assessed via a countermovement jump (CMJ) on a dual force platform (ForceDecks FD4000, Vald Performance, Australia), with raw data recorded at 1000 Hz. Prior to testing, players completed a 10-min dynamic warm up consisting of foam rolling, active mobility, and practice jumps. For a successful test, players were required to keep their hands in contact with their hips for the duration of the movement and land whist maintaining balance. Players were instructed to jump as high as they could for three repetitions separated by a short pause of ~3 s to maintain balance and prepare to jump again. The average peak power, the flight time:contraction time ratio (FT:CT) and the peak rate of force development (PRFD) were then identified for further analysis. The average peak power was normalised by dividing the measures by body mass. A previous study reported low between-session measurement error (coefficient of variation) for these measures and the protocol, with coefficients of variation ranging from 3.38 to 4.47% (Petre et al., 2023). Upper body neuromuscular performance was assessed via a bench throw test on a Smith machine. Prior to testing, players completed a 10-min warm-up consisting of active mobility and muscle activation exercises. The same warm-up activities were performed by each athlete to ensure standardisation, and to minimise the risk of injury. To complete the test, players laid supine on a bench and were instructed to push a loaded barbell (total weight = 30 kg) as high as possible with two hands for three consecutive attempts separated by a short pause of at least 2 s. The bench throw test was conducted using 30 kg as this load was reported to detect subtle changes in response to an intervention compared to other loads (Loken et al., 2021). Average peak power (BT-PP) was calculated using a linear position transducer (GymAware PowerTool, GymAware, Canberra, Australia) attached to the barbell.

### Statistical Analysis

The SPSS statistical software package (SPSS inc., Chicago, IL, USA) was used to complete a two-way (week x day) repeated measures analysis of variance for all variables. According to the Shapiro-Wilk test, the majority (70%) of the data was normally distributed. Where appropriate, a Bonferroni’s corrected, pairwise comparison, post-hoc test was completed to identify statistical differences between factors and a *p* value of less than 0.05 was considered statistically significant. Effect size with the associated 95% confidence interval was calculated to compare the magnitude of differences between weeks for main effects, using the formula for a repeated measures design (Uanhoro, 2017). Given the main week effects, correlation coefficients were unable to be calculated for effect size. Thus, the correlation coefficient was set at 0.5 for all measures, which is considered to be conservative when dealing with missing data (Baumert et al., 2016). For effect size calculations, the values of ≤0.2, ≥0.5 and ≥0.8 were considered small, moderate and large, respectively (Cohen, 1988).

## Results

### Sample Size

Due to technical difficulties during data collection, the results for each variable consisted of between 16 and 18 athletes. The sample size for each variable is provided in tables and figures. Furthermore, missing data (<3% of the total sample) were evident, and thus were filled by averaged values from the sample of the affected time points (Pierce et al., 2018).

### Creatine Kinase Activity

**Figure 2:**
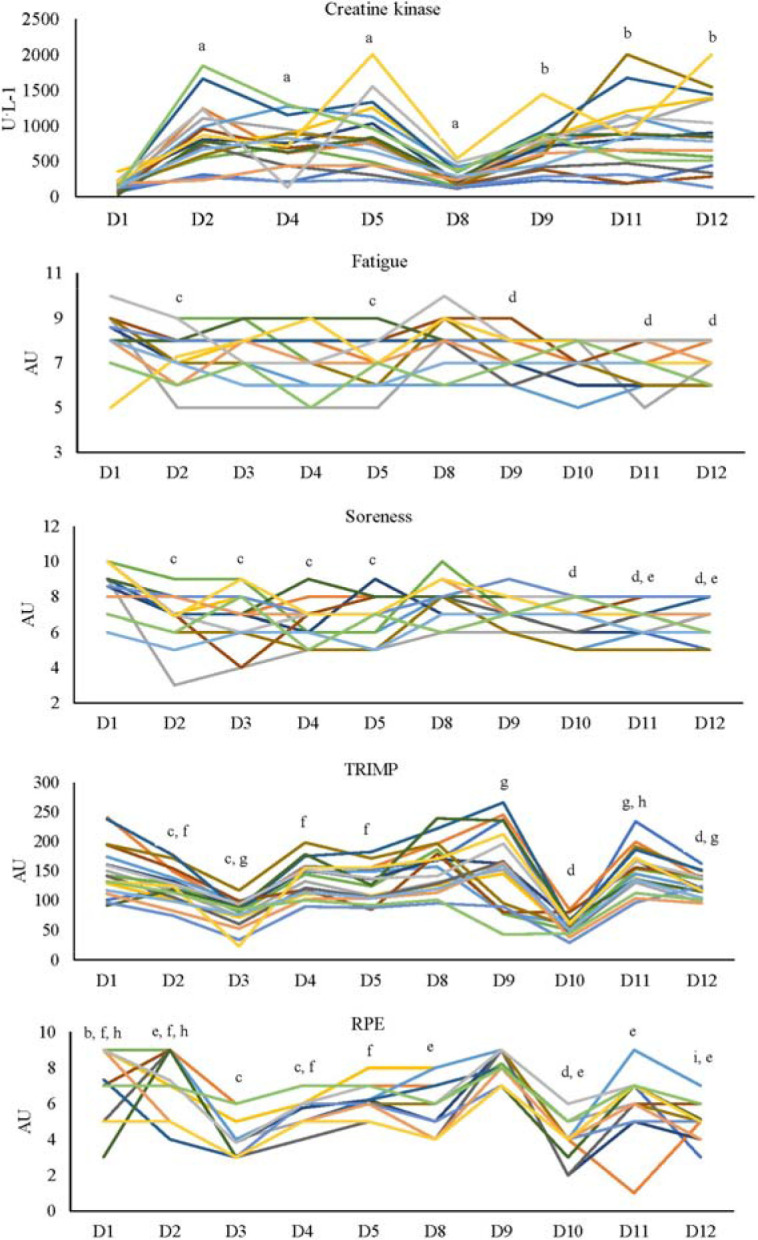
Intra-individual variation of creatine kinase and internal training loads from day 1 to day 12 across the two weeks of training. *a: greater than D1 (p < 0.05); b: greater than D8 (p < 0.05); c: lower than D1 (p < 0.05); d: lower than D8 (p < 0.05); e: lower than D9 (p < 0.05); f: greater than D3 (p < 0.05); g: greater than D10 (p < 0.05); h: greater than D4 (p < 0.05); i: lower than D5 (p < 0.05) TRIMP: training impulse recorded from the heart rate; RPE: rating of perceived exertion; AU: arbitrary units Sample size: n = 18 for all measures*

A significant interaction effect was identified for CK activity (*p* = 0.01). Post-hoc analysis showed that CK activity was significantly greater during D2–D5 compared to D1 in week 1, with the same trends observed in week 2 (Figure 2). There were no differences in CK activity between weeks (*p* = 0.63, i.e., no main effect of the week). However, a main effect of the day was evident (*p* < 0.001) with significantly lower activity on the first day (*p* < 0.001) compared to all other testing days (Table 1).

### Internal Training Loads

There was a significant interaction effect for the TRIMP (*p* = 0.01), the sRPE (*p* < 0.001) and soreness (*p* = 0.02), but not for perceived fatigue (*p* = 0.75) (Figure 2). The TRIMP on D3 was significantly greater than on D10 (*p* < 0.001), whilst on D11 it was significantly greater than on D4 (*p* = 0.03). However, there was no difference between weeks as a main effect (*p* = 0.27). Furthermore, the TRIMP on D1 was significantly greater than on D2 (*p* = 0.005) and D3 (*p* < 0.001) and on D2 it was significantly greater than on D3 (*p* < 0.001) in week 1. However, only on D10 it was significantly lower than on D8, D9, D11 and D12 (*p* < 0.001) in week 2. Soreness was significantly greater on D1 than D8 (*p* = 0.008), although there was no difference between weeks as a main effect (*p* = 0.65). Within each week, soreness was significantly greater on D2–D5 than D1 (*p* < 0.01) in week 1 (Figure 2), and similar trends were identified in week 2. When compared between weeks, the RPE on D9 and D12 was greater than on D2 (*p* = 0.04) and D5 (*p* < 0.001), whilst on D1 it was greater than on D8 (*p* < 0.001; Figure 2). However, there was no difference between weeks as a main effect (*p* = 0.30). As a main effect of the day, the RPE was lower on D3 (*p* < 0.001) and D5 (*p* = 0.047) compared to D1 (Table 1).

**Table 1:** The mean ± standard deviation of EIMD, internal and external training loads, and neuromuscular function metrics for each day of the week.

	First day of the week (D1 and D8)	Second day of the week (D2 and D9)	Third day of the week (D3 and D10)	Fourth day of the week (D4 and D11)	Fifth day of the week (D5 and D12)
**EIMD**					
Creatine Kinase (U·L^−1^; n = 18)	209.5 ± 90.4	776.5 ± 314.4^a^	-	781.4 ± 348.7^a^	878.8 ± 447.2^a^
**Internal Training Load**					
Fatigue (au; n = 18)	8.3 ± 0.8	7.4 ± 0.8^a^	7.4 ± 0.7^a^	7.1 ± 0.9^a^	7.0 ± 0.8^a^
Muscle Soreness (au; n = 18)	8.3 ± 0.9	7.1 ± 0.9^a^	6.8 ± 1.0^a^	6.7 ± 0.8^a^	6.7 ± 0.8^a^
TRIMP (au; n = 18)	153.9 ± 36.5	139.7 ± 38.2	65.9 ± 16.1^a,b,d,e^	146.0 ± 30.5	125.2 ± 22.5^a,c,d^
sRPE (au; n = 18)	6.5 ± 1.4	7.8 ± 1.1^a^	4.0 ± 0.9^a,b,d,e^	6.0 ± 0.8^b^	5.6 ± 0.8^a,b^
**External Training Load**					
Total Distance (m; n = 16)	4564.1 ± 423.2	5048.9 ± 194.4^a^	2207 ± 231.2^a,b,d,e^	5131.9 ± 239.6^a^	4704.4 ± 157.6^b,d^
High-Velocity Distance (m; n = 17)	527.2 ± 155.9	973.1 ± 101.4^a^	267.6 ± 49.1^a,b,d,e^	554.5 ± 116.7^b^	597.1 ± 103.9^b^
RHIE (au; n = 17)	13.7 ± 2.2	17.1 ± 2.6^a^	11.2 ± 2.2^a,b,d,e^	15.3 ± 2.3	17.9 ± 2.1^a,d^
Player Load (au; n = 17)	514.7 ± 45.8	576.7 ± 40.4^a^	271.3 ± 34.2^a,b,d,e^	583.3 ± 49.9^a^	545.1 ± 42.1^b,d^
Impacts (frequency; n = 18)	3.6 ± 1.6	6.1 ± 3.8	4.9 ± 3.8	5.4 ± 3.7	11.8 ± 4.8^a,b,c,d^
Strength Volume (kg; n = 18)	21,042.4 ± 6357.2	-	6984.0 ± 1506.6^a^	-	12,423.0 ± 4266.8^a,c^
**Neuromuscular Function**					
CMJ Peak Power (W·kg^−1^; n = 17)	55.9 ± 7.4	-	-	-	53.6 ± 6.6^a^
CMJ FT:CT (n = 17)	0.8 ± 0.1	-	-	-	0.7 ± 0.1^a^
CMJ PRFD (N·s^−1^; n = 17)	1859.9 ± 1542.9	-	-	-	1935.9 ± 1200.6
Bench Throw Peak Power (W·kg^−1^; n = 18)	12.1 ± 1.0	-	-	-	12.1 ± 1.4

Values are reported as mean ± SD. EIMD = exercise induced muscle damage; TRIMP = training impulse; sRPE = session rating of perceived exertion; RHIE = repeat high-intensity efforts; CMJ = countermovement jump; FT = flight time; CT = contraction time; PRFD = peak rate of force development; U/L = units per litre; au = arbitrary units; W = watts; n = sample; m = meters; kg = kilograms; N = newtons; s = seconds: a = p < 0.05 vs. first day of the week; b = p < 0.05 vs. second day of the week; c = p < 0.05 vs. third day of the week; d = p < 0.05 vs. fourth day of the week; e = p < 0.05 vs. fifth day of the week

### External Training Loads

**Figure 3:**
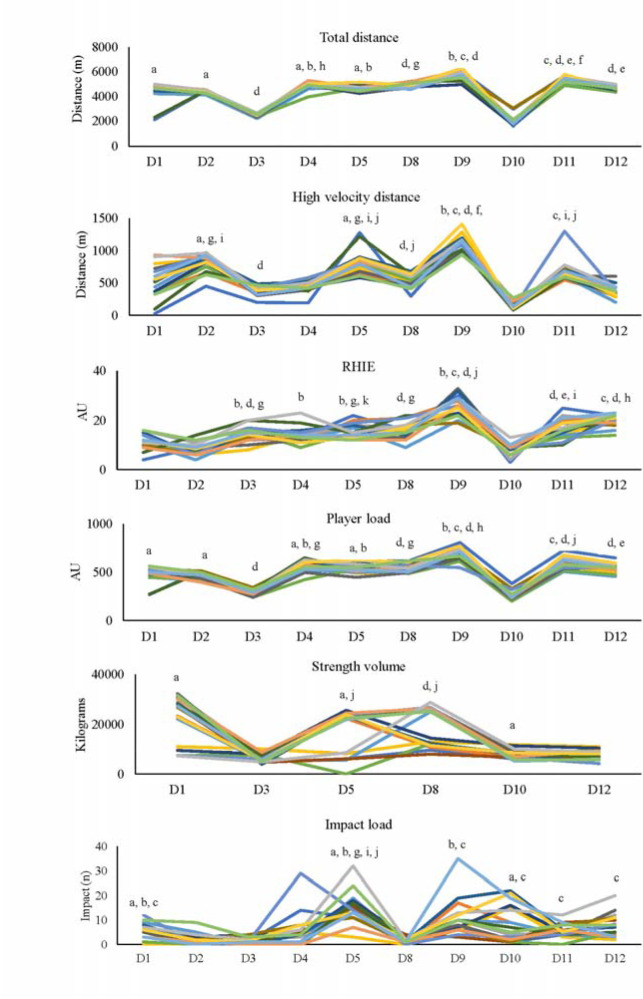
Intra-individual variation of external load metrics from day 1 to day 12 across the two weeks of training. a: greater than D3 (p < 0.05); b: greater than D2 (p < 0.05); c: greater than D8 (p < 0.05); d: greater than D10 (p < 0.05); e: lower than D9 (p < 0.05); f: greater than D11; g: greater than D1 (p < 0.05); h: lower than D11 (p < 0.05); i: greater than D4 (p < 0.05); j: greater than D12 (p < 0.05); k: lower than D12 (p < 0.05) RHIE: repeated high intensity efforts; AU: arbitrary units Sample size: Total distance (n = 16); High velocity distance (n = 17); RHIE (n = 17); Player load (n = 17); Strength volume (n = 18); Impact load (n = 18)

A significant interaction effect was found for all external training load measures (*p* < 0.001). Post-hoc analysis identified a significant increase for numerous measures (total distance, high-velocity distance, RHIE, player load and impacts) on most days in week 2 compared to week 1 (Figure 3). However, strength volume was greater on D10 (week 2) than D3 (week 1), whilst on D5 (week 1) it was greater than on D12 (week 2). There was a main effect of the week (*p* < 0.01) for most external training load measures with significantly greater values for total distance, RHIE and PlayerLoadTM during week 2 compared to week 1 (Table 2). However, strength volume was significantly greater during week 1 compared to week 2 (*p* = 0.008). There was a main effect of the day for all external measures, with total distance, RHIE, PlayerLoadTM and impact on most days greater than on D1 (*p* < 0.05; Table 1). However, strength volume was greater on D3 and D5 than D1 (*p* < 0.001), and high velocity distance was greater on D4 and D5 than D1 (*p* < 0.05; Table 1).

### Neuromuscular Function

None of the neuromuscular function measures showed significant interaction effects (*p* = 0.40). However, there was a significant decline in lower body neuromuscular function (i.e., CMJ-RPP, *p* = 0.002 and CMJ-FT:CT, *p* = 0.01) in week 2 as a main effect of the week (Table 2), although there was no difference between weeks for the CMJ-PRFD (*p* = 0.06). Furthermore, there was an increase in upper body neuromuscular power output (i.e., bench throw relative peak power, *p* = 0.03) compared to week 1 (Table 2). There was a main effect of the day (*p* < 0.001) for the CMJ-RPP and the CMJ-FT:CT (*p* < 0.01), which was reduced on the fifth day relative to the first day of the week (Table 1), although there were no differences between these days for the CMJ-PFRD (*p* = 0.64) and BT-PP (*p* = 0.95).

**Table 2:** The mean ± standard deviation of EIMD, internal and external training loads, and neuromuscular function metrics for each week of the study.

	Week 1	Week 2	Effect size (95% CI)
**EIMD**			
Creatine Kinase (U·L^−1^; n = 18)	649.2 ± 255.0	673.8 ± 299.1	−0.08 (–0.56, 0.38)
**Internal Training Load**			
Fatigue (au; n = 18)	7.5 ± 0.7	7.4 ± 0.7	0.14 (–0.32, 0.61)
Muscle Soreness (au; n = 18)	7.2 ± 0.8	7.1 ± 0.8	0.12 (–0.34, 0.59)
TRIMP (au; n = 18)	123.4 ± 25.9	128.9 ± 28.4	–0.19 (–0.67, 0.27)
sRPE (au; n = 18)	6.1 ± 0.8	5.9 ± 0.8	0.24 (–0.22, 0.72)
**External Training Load**			
Total Distance (m; n = 16)	4137.8 ± 198.4	4525.0 ± 169.2^a^	–1.98 (–2.98, –1.20)*
High-Velocity Distance (m; n = 17)	596.6 ± 84.1	571.1 ± 58.5^a^	0.33 (–0.16, 0.85)
RHIE (n; n = 17)	12.6 ± 1.8	17.5 ± 1.8^a^	–2.59 (–3.76, –1.67)*
Player Load (au; n = 17)	470.9 ± 31.7	525.6 ± 43.7^a^	–1.33 (–2.11, –0.73)*
Impacts (frequency; n = 18)	5.9 ± 2.6	6.8 ± 3.0	–0.30 (–0.79, 0.16)
Strength Volume (kg; n = 18)	15369.7 ± 4885.4	11597.3 ± 2594.8^a^	0.85 (0.36, 1.55)*
**Neuromuscular Function**			
CMJ Peak Power (W·kg^−1^; n = 17)	55.4 ± 7.0	54.1 ± 7.0^a^	0.18 (–0.30, 0.66)
CMJ FT:CT (n = 17)	0.8 ± 0.1	0.7 ± 0.1^a^	0.95 (0.40, 1.58)*
CMJ PRFD (N·s^−1^; n = 17)	2212.5 ± 1905.7	1583.4 ± 884.8	0.36 (–0.13, 0.97)
Bench Throw Peak Power (W·kg^−1^; n = 18)	11.8 ± 1.2	12.3 ± 1.2^a^	–0.39 (0.90, 0.07)

Values are reported as mean ± SD. EIMD = exercise induced muscle damage; TRIMP = training impulse; sRPE = session rating of perceived exertion; RHIE = repeat high-intensity efforts; CMJ = countermovement jump; FT = flight time; CT = contraction time; PRFD = peak rate of force development; U/L = units per litre; au = arbitrary units; W = watts; n = sample; m = meters; kg = kilograms; N = newtons; s = seconds; CI – confidence interval: a = p < 0.05 vs. week 1. * Large effect size

## Discussion

The aims of this study were to identify the physiological responses to the initial two weeks of the preseason phase in elite male RL athletes and determine whether there was an occurrence of an RBE. The present findings identified similar CK activity, muscle soreness and internal training loads across the initial two weeks of the preseason in elite male RL athletes. A significant increase in numerous external training load variables was identified in the second week of the study, while there was a decline in lower body neuromuscular function. Despite a significant increase in external training loads from week 1 to week 2, similar EIMD and internal training loads were exhibited. These findings can be used by practitioners to inform the training prescription in the initial weeks of the preseason.

The key finding of the current study was the lack of change in CK activity, muscle soreness and internal training loads, concurrently with a significant increase in external training loads during the early preseason. These findings support a recent review (Simmons et al., 2021), which identified no change in CK markers in response to repeated bouts of a given training stimulus in elite and semi-elite athletes. The theoretical construct of the RBE suggests that CK activity and muscle soreness will reduce following repeated exposure to the same training stimulus (McHugh et al., 1999; Simmons et al., 2021). Although the mechanisms resulting in this attenuation of CK markers and muscle soreness are not fully understood, it has been suggested that neural, cellular and connective tissue adaptations may all contribute to this RBE (McHugh et al., 1999). It could be concluded that athletes within the current study did not demonstrate the typical responses associated with the theoretical RBE as there were no significant reductions in CK activity and muscle soreness from week 1 to week 2. Whilst the current findings do not support this traditional definition of the RBE, the work of Burt and colleagues (2015) provides an alternate suggestion for an RBE.

They identified similar CK activity and muscle soreness concurrently with an increase in training loads as well as a maintenance of training loads across two resistance training bouts (Burt et al., 2015). Those authors indicated that CK activity and muscle soreness attenuations were not dependent upon alterations in workloads (Burt et al., 2015). Similarly, athletes in the current study were exposed to a significant increase in external training loads in week 2 yet, maintained a CK activity response similar to that of week 1. Potentially, the current athletes exhibited similar levels of CK markers and perceptual measures for both weeks due to neural, cellular and connective tissue adaptations underpinning the RBE (McHugh et al., 1999). Traditionally, the RBE phenomenon has been confirmed in previous studies by incorporating identical resistance training bouts, interspersed by approximately 2–3 weeks, under a highly controlled, laboratory condition (Doma et al., 2015, 2017). However, athletes rarely undertake identical micro-cycles during the preseason, given the importance of applying progressive overloads and limiting the possibility of diminishing returns (Jeong et al., 2011). Thus, the acute responses reported in the current study reflect ‘real world’ training situations in elite male RL players during the early weeks of the preseason. These results, along with previous research (Burt et al., 2015) could provide the foundation for an alternate view of the RBE.

An important and novel element of the current study was the inclusion of physical performance testing throughout the two training micro-cycles of the preseason. A previous systematic review (Simmons et al., 2021) identified a lack of regular physical performance testing in prior work, which is essential in identifying training-induced physical performance changes. Multiple studies (Coutts et al., 2007b; Miloski et al., 2016; Silva et al., 2014) have included inconsistent physical performance testing regimes within micro-cycles with testing completed every couple of weeks or months. Conducting physical performance tests over extended periods does not capture the acute changes in physical performance capabilities due to training-induced stress (e.g., increased CK activity or muscle soreness). Several studies (Coutts et al., 2007b; Miloski et al., 2016; Silva et al., 2014) have reported improvement in physical performance measures (e.g., CMJ) across several weeks and months, indicating chronic rather than acute adaptations. To our knowledge, the current study was the first to examine acute responses in physical performance capabilities across two successive micro-cycles of a preseason in elite RL players with increasing external training loads reflecting authentic periodisation. This regular assessment may provide a less invasive method of monitoring EIMD in elite team sport athletes.

The findings of this study identified a significant decline in two of the lower body neuromuscular function metrics across the weeks. The significant reduction in CMJ performance in week 2 highlighted a negative acute response with attenuation of muscle contractility, which is a common symptom of EIMD (Warren et al., 1999). Both resistance training and field-based sessions involved numerous eccentric contractions, which are known to induce greater EIMD and chronic muscular adaptations (Hedayatpour and Falla, 2015). Although the biweekly testing of neuromuscular function in this study demonstrated an acute reduction in physical performance capabilities, this decrement in neuromuscular function may be required for the achievement of positive chronic adaptations. Future research could longitudinally observe these weekly responses to modify the training prescription and further promote physiological adaptations.

The current increases in CK activity, muscle soreness and fatigue (i.e., internal training loads) confirmed previous findings observed in the initial phase of the preseason period in semi-elite populations (Coutts et al., 2007a, 2007b; Miloski et al., 2016). To our knowledge, the current study is the first to assess repetitively CK activity, muscle soreness and fatigue throughout the initial micro-cycles of the preseason phase in team sport athletes. This consistent monitoring was able to detect a sustained increase in internal response across the two micro-cycles at a time of an external training load increase relative to the preceding off-season period (Simmons et al., 2021). Furthermore, this regular monitoring was able to identify a decline in lower body neuromuscular function on the fifth day of the week indicating a potential compromise in muscular contractility (Doma et al., 2015). Practitioners should be aware of the heightened physiological response in this early phase of the preseason macro-cycle and ensure training programs promote adaptation without exposing athletes to the risk of overtraining. Incorporating effective recovery regimes may adequately prepare athletes for future training stimuli, enhance training quality and optimise adaptation (Doma et al., 2017).

Given the sharp increase in the external training load from the off-season to the initial weeks of the preseason period, practitioners should complete daily monitoring of external and internal training loads in collision sport athletes. Understanding each athletes’ off-season training load would also aid in prescribing training with the goal of gaining maximum training adaptations. Although practitioners may not observe an initial preseason reduction in CK activity and muscle soreness (i.e., internal training loads) in response to an increase in external training loads, athletes may still be adapting to the training stimulus. This study provided a ‘real world’ example of how elite RL athletes respond to the early phase of the preseason period.

Although this study identified key findings concerning the initial phase of the preseason, there were some limitations relevant for practitioners. Firstly, due to economic feasibility and the invasive nature of CK collection, this study investigated the initial two weeks of the preseason only. Although this is an important period for practitioners to monitor, future research could potentially investigate the entire preseason and validate other non-invasive markers of EIMD (e.g., delayed onset muscle soreness) for greater monitoring. Secondly, the external training load of athletes in the weeks preceding the study period were unknown. Due to the unstructured nature of the off-season phase, it is possible that athletes participated in varying types and volumes of training. Although it did not affect baseline levels, this may have impacted the magnitude of CK activity, muscle soreness and adaptation to the structured training stimuli. Although quantifying training loads in the off-season phase is not a common practice in elite RL, practitioners should be aware of any off-season training undertaken by athletes and potentially alter the training prescription in the initial micro-cycles of the preseason to optimise adaptation.

## Conclusions

In conclusion, CK activity and muscle soreness did not change in response to an increase in external training loads across the initial two weeks of the preseason period in elite male RL athletes. Although these findings do not support the traditional definition of the RBE, they may provide a foundation for a ‘real world’ perspective of the phenomenon that may be more applicable for team sport practitioners.
